# The Effects of Virtual Reality in Targeting Transdiagnostic Factors for Mental Health: A Systematic Review of the Literature

**DOI:** 10.3390/jcm11216463

**Published:** 2022-10-31

**Authors:** Valentina Gardini, Giorgia Gamberini, Sophia Müller, Silvana Grandi, Elena Tomba

**Affiliations:** 1 Department of Psychology, University of Bologna, 40127 Bologna, Italy; 2 Institute for Psychology, Department of Clinical Psychology, University of Bern, 3012 Bern, Switzerland

**Keywords:** virtual reality, transdiagnostic factors, avoidance, emotion regulation, cognitive reappraisal, aggression, impulsivity

## Abstract

Virtual reality (VR) was found to be effective in the treatment of several specific psychopathologies. However, the effects of VR-based interventions beyond the disorder-specific approach and their ability to improve transdiagnostic factors of mental disorders are unknown. This PRISMA systematic review was conducted using databases PubMed and PsycINFO, searching articles published between 2010 and September 2022. Keywords “emotion regulation”, “cognitive reappraisal”, “avoidance”, “impulsivity”, “aggression”, and “disinhibition” were combined with “virtual reality” to retrieve studies showing the effects of VR-based interventions on these transdiagnostic factors. 29 experimental studies and seven case-studies were selected. A total of 23 considered avoidance, eight dealt with emotion regulation, three concerned aggression, two addressed impulsivity, two dealt with cognitive reappraisal, and none examined disinhibition. Most of the studies included anxiety disorder patients (*n* = 15), especially with specific phobias (*n* = 8) and social anxiety disorder (*n* = 4). VR managed to improve all transdiagnostic factors, with results often maintained at follow-ups (*n* = 21 studies; range: 1–12 months) and similar to traditional interventions (e.g., cognitive-behavioral therapy). Exploring the transdiagnostic potential of VR may help to reduce costs and improve applicability in clinical psychology. While results were promising, further studies are needed for aggression, impulsivity and cognitive reappraisal, especially including follow-ups, comparisons with first-line treatments, and understudied clinical populations.

## 1. Introduction

In recent years, virtual reality (VR) has emerged as a new tool to assist clinicians in the treatment of several psychiatric disorders, especially in anxiety, psychotic, substance-related, and eating disorders [1,2] because of its ability to provide a systematic and controlled exposure therapy without the complications of in vivo exposure [3]. Moreover, VR can also be used in association with cognitive-behavioral treatments (CBT) and other psychotherapeutic interventions such as mindfulness-based cognitive therapy [4] and dialectical behavioral therapy [5] that are designed to improve existing treatment protocols for several psychiatric disorders (e.g., anxiety disorders, eating disorders, substance-use disorders, psychosis, etc.) [1].

Nonetheless, several downsides in relation to VR have also been reported that make its application difficult in the clinical setting, such as its high cost [6] and the need to have different software for specifically tackling different psychiatric disorders. Indeed, the disorder-specific approach that has been predominantly used to design VR software and interventions is also the approach that is most widely used for traditional CBT. CBT interventions, in fact, are usually designed for the treatment of one single disorder at a time [7,8,9], since they are based on assumptions coming from the conventional nosological systems, such as the DSM-5 and the ICD-10 [10,11]. These diagnostic systems view psychopathologies as distinct, independent, and categorical constructs, with patients either meeting or not the diagnostic criteria for a specific psychiatric disorder [12].

Things, however, are much more complicated than this in the clinical experience, where often a person can present more than one psychiatric disorder in comorbidity.

Recently, clinical research in fact turned to the transdiagnostic model to explore mental disorders and overcome the limits of a disorder-specific approach [13]. The transdiagnostic framework emphasizes in particular that mental health disorders share common underlying psychological factors, or transdiagnostic factors [14], which when targeted simultaneously may allow for the treatment of both the main disorder and its comorbidities [9,15]. Thus far, while the literature has no accordance about a shared list of transdiagnostic factors that can cause and maintain several psychological disorders, the internalizing-externalizing model of psychopathology is one of the most widely accepted transdiagnostic models [16]. In the model, transdiagnostic factors are divided into two main categories: internalizing factors, which include several over-inhibited or internally-focused symptoms (e.g., avoidance, negative emotions, social withdrawal, somatic complaints, etc.), and externalizing factors, which in turn include disinhibited or externally-focused behavioral symptoms (e.g., aggression, impulsivity, disinhibition, conduct problems, delinquent behavior, oppositionality, hyperactivity, attention problems, etc.). Subsequently, transdiagnostic interventions based on CBT therapy have been developed to target these specific internalizing and externalizing transdiagnostic factors such as the unified protocol, the shared mechanisms treatments, and transdiagnostic cognitive-behavioral therapy [17]. More specifically, the Unified Protocol for Transdiagnostic Treatment of Emotional Disorders [18] is the most widely studied evidence-based protocol [19], which proved to be effective for the treatment of anxiety and mood disorders [20,21], with results similar to disorder-specific treatments [22,23].

Since many authors agree that transdiagnostic treatments would be more advantageous than disorder-specific treatments for their ability to increase cost efficiency [9,24], if transposed to the development of VR software the transdiagnostic approach might be able to also overcome the limits (in terms of costs and training) of this technology in the field of clinical psychology.

In order to understand the transdiagnostic potential of VR, this systematic review of the literature aims to explore whether some of the main internalizing and externalizing transdiagnostic factors for mental health disorders [12,14] can be improved by VR-based treatments.

## 2. Materials and Methods

### 2.1. Protocol and Search Strategy

This systematic review has been conducted accordingly to the Preferred Reporting Items for Systematic Reviews (PRISMA) criteria guidelines [25] and was not registered on a public repository. Two databases (PubMed and PsycINFO) were used to retrieve articles for the present review: authors selected six keywords to identify some of the main internalizing and externalizing transdiagnostic factors for mental health disorders [12,14]: “avoidance”, “aggression”, “disinhibition”, “emotion regulation”, “reappraisal”, and “impulsivity”. In particular, due to the lack of a set list of transdiagnostic factors in the literature, the authors decided to select clinically relevant transdiagnostic factors that have been found to be present and play a role in many psychopathologies. For example, different types of avoidance (e.g., avoidance behaviors, social avoidance, experiential avoidance, cognitive avoidance, etc.) characterize a wide variety of mental health disorders, such as mood and anxiety disorders, post-traumatic stress disorders (PTSD), psychotic disorders, and obsessive-compulsive disorders [26,27,28,29]. Similarly, difficulties in using emotion regulation strategies and cognitive reappraisal are linked to the maintenance and development of several psychopathologies and represent some of the main targets of transdiagnostic CBT-based treatments [15,30,31,32]. Finally, aggressive behaviors, impulsivity, and disinhibition can also be found in diagnostic criteria and clinical presentations of different psychiatric diagnoses and especially externalizing disorders (e.g., substance-use disorders, antisocial personality disorder, attention-deficit-hyperactivity disorder, etc.) [10,11,12,33,34,35,36].

All of the keywords identifying these transdiagnostic factors were combined with the keyword “virtual reality” using the Boolean operator “AND”.

During the database search, results were filtered for English and Italian language, journal articles, clinical trials (PubMed), and academic journals (PsycINFO). With regard to publication dates, the authors selected a range between 2010 and September 2022. Duplicate articles generated across databases were identified and excluded.

Titles and abstracts were screened by two authors (G.G., S.M.) in order to asses which ones would fulfill the aforementioned aims of the review. Articles that appeared potentially eligible for the review were retrieved and reviewed by two authors (V.G., G.G.), who independently assessed each of the full reports, arriving at a consensus regarding eligibility. When disagreements between the two authors arose, multiple rounds of full-text revision and discussions were undertaken until consensus was reached, with the involvement of a third author (E.T.) when needed. 

**Figure 1:**
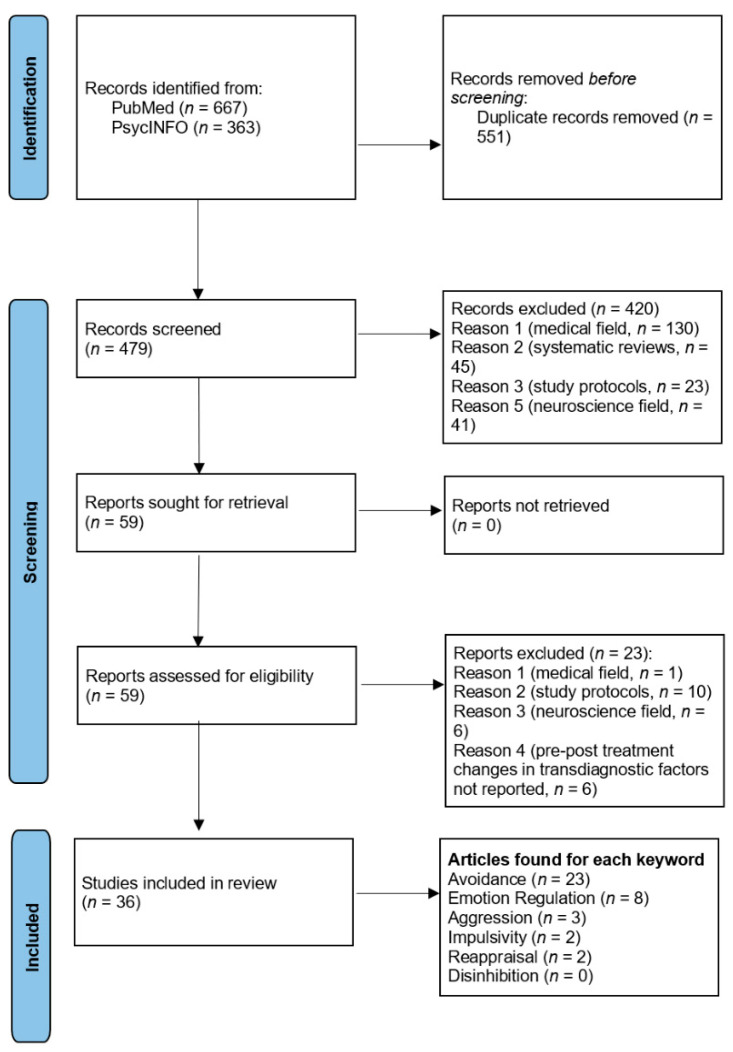
PRISMA flow diagram summarizing the article selection process [25].

A visual representation of the article selection process is presented in the PRISMA flow diagram (Figure 1) below.

### 2.2. Eligibility Criteria 

Articles were considered eligible for the review if they were randomized controlled trials (RCTs), longitudinal studies, or case studies evaluating changes in patients’ levels of the considered transdiagnostic factors between pre- and post- VR-based intervention. Study protocols, dissertations, systematic and non-systematic reviews, meta-analyses, medical or neuroscience studies and books (or book chapters) were excluded from the research. More information about inclusion and exclusion criteria for articles selection are summarized in the Population, Intervention, Comparison, Outcomes and Study (PICOS) table below (Table 1) [37]. 

### 2.3. Data Extraction

Following the database search, duplicate articles generated across the two databases (PubMed and PsycINFO) were selected and removed. The authors then proceeded to screen titles and abstracts of the articles and excluded those which did not seem relevant to the keywords and aims listed above. All of the remaining articles were subsequently read thoroughly in order to examine whether changes in the aforementioned transdiagnostic factors following a VR-based intervention were assessed between pre- and post- treatment. When present, data regarding follow-ups were also collected. Data extraction was performed independently by two of the authors (G.G. and V.G.). The authors followed the PICOS pre-set extraction criteria (see Table 1) and systematically summarized the relevant data of each article in a separate table (Table S1). 

### 2.4. Quality and Risk of Bias Assessment 

Quality and risk of bias assessment was conducted using a customized checklist retrieved from the National Institutes of Mental Health’s tool (2021) [38] on experimental articles only, while this assessment was not conducted on case studies. See Table S2 for criteria.

## 3. Results

### 3.1. Results of Literature Search

A total of 1030 articles were retrieved from PubMed and PsycINFO. After the removal of 551 duplicates (53.50%) and the exclusion of 420 articles (40.78%) (see Figure 1), a total of 59 articles (5.73%) were considered potentially eligible for the review. After full-text screening of the articles, 36 articles (3.50%; *n* = 29 experimental studies and *n* = 7 case studies) were finally included in the review.

The number of studies taken into consideration for each transdiagnostic factor varied. Avoidance was the most studied transdiagnostic factor found in 23 articles (63.89%) (*n* = 19 experimental studies and *n* = 4 case studies) [39,40,41,42,43,44,45,46,47,48,49,50,51,52,53,54,55,56,57,58,59,60,61], followed by emotion regulation, which was found in eight articles (22.22%; *n* = 7 experimental studies and *n* = 1 case study) [62,63,64,65,66,67,68,69]; aggression, which was found in three experimental studies (8.33%) [70,71,72]; impulsivity, which was found in two studies (5.56%; *n* =1 experimental study and *n* = 1 case study) [71,73]; and cognitive reappraisal, which was found in two studies (5.56%; *n* = 1 experimental study and *n* = 1 case study) [63,74]. No studies were found for the transdiagnostic factor of disinhibition. Moreover, two studies took into consideration two transdiagnostic factors simultaneously: emotion regulation and reappraisal [63], and aggression and impulsivity [71].

### 3.2. Characteristics of the Studies 

Among the 36 articles that were selected for the review, different sample sizes were used. Sixteen experimental studies (44.44%) had less than 50 participants [40,44,45,47,48,50,51,54,58,60,62,63,66,67,68,72], nine (25%) included between 50 and 100 participants [39,46,49,53,55,59,64,69,70], and only a few (*n* = 4, 11.11%) had a sample size larger than 100 participants [43,52,56,71]. Finally, seven (19.44%) articles were multiple or single case studies including between one and eight participants [41,42,57,61,65,73,74].

In terms of research design, only 23 out of 36 studies (63.89%) were controlled studies, of which 19 were randomized controlled trials (RCTs) (82.61%) [39,43,45,46,48,49,50,51,53,54,55,56,59,60,63,64,66,68,71] and four (17.39%) were non-randomized controlled studies [47,67,69,70]. Six studies out of 36 (16.67%) were uncontrolled clinical trials [40,44,52,58,62,72], and seven (19.44%) were case studies [41,42,57,61,65,73,74]. A total of 21 studies (58.33%) also included a follow-up (ranging from 1 to 12 months) [39,42,43,44,45,46,47,49,51,54,55,56,58,59,60,61,63,64,71,72,73].

Regarding sociodemographic characteristics of the samples in the reviewed studies, the age range went from young children to adults (around 8 to 51 years of age). However, the great majority of the studies were carried out on adult samples between ~19 and 51 years of age (*n* = 31, 86.11%), with only two studies (5.56%) carried out on children between ~8 and 10 years of age [42,69], and three (8.33%) on adolescents between ~12 and 15 [64,68,74] Moreover, mixed gender was predominant in the studies (*n =* 23; 36.11%), with some exceptions: four studies (11.11%) had an all-male sample [41,70,72,74], and four (11.11%) had an all-female sample [57,61,65,73]. Moreover, five studies (13.89%) did not clearly state the participants’ gender [44,49,53,60,71].

Lastly, regarding the kind of VR technology that was used among all 36 studies, 28 (77.78%) used immersive VR [39,40,41,42,43,44,45,46,48,49,50,51,52,54,55,56,58,59,60,61,62,64,65,66,67,68,70,71], 3 (8.33%) Cave Automatic Virtual Environment (CAVE) [53,69,73], and only five (13.89%) used non-immersive VR technology [47,57,63,72,74]. See Table S1 for characteristics of the studies and the specific contents, data and results of the selected papers.

### 3.3. Clinical and Non-Clinical Populations Included in the Studies

The majority of the studies (*n* = 17; 47.22%) carried out on adults focused on clinical populations. Patients with anxiety disorders were the most frequent population involved in the studies (*n* = 15, 41.67%), of which eight (22.22%) included patients with specific phobias [41,44,45,47,54,55,60,61], four (11.11%) included patients with social anxiety disorder (SAD) [39,46,49,59], and three (8.33%) included patients with panic disorder and agoraphobia [50,51,53]. Patients with PTSD [40,52,72] and psychotic disorders [43,56,58] were the second most found clinical populations, appearing in three (8.33%) studies each. Other less studied clinical populations included eating disorders (*n* = 1, 2.78%) [57], borderline personality disorders (*n* = 1, 2.78%) [54], OCD (*n* = 1, 2.78%) [73] and forensic patients (*n* = 1, 2.78%) [71]. Four (11.11%) studies, instead, were carried out on the general population or on healthy adults [62,63,67,70].

In the studies carried out on children (*n* = 2, 5.56%), one (2.78%) was carried out on children with specific phobias (dogs) [42] and one (2.78%) on children with autism spectrum disorder [69]. Adolescent samples in the studies, instead, were taken from the general population (*n* = 2, 5.56%) [64,68] or involved teens with acute anxiety, suicidal thoughts and low mood [74] (see Table S1 for additional details of each study).

### 3.4. Quality and Risk of Bias Assessment

Quality and risk of bias assessment was performed only on experimental studies. As shown in Table S3, the quality of the selected 29 experimental studies widely differed. In particular, 15 (51.72%) were ranked as having a strong quality [39,43,45,46,51,53,54,55,56,58,59,60,64,71,72], eight (27.59%) were ranked as having moderate quality [44,47,48,49,52,66,68,70], and six (20.69%) were ranked as weak [40,50,62,63,67,69]. A methodological issue often found throughout the reviewed studies was the absence of a follow-up: 11 (37.93%) out of the 29 experimental studies did not include follow-up for the selected outcomes [40,48,50,52,53,62,66,67,68,69,70].

### 3.5. Results about the Effects of Virtual Reality-Based Interventions on Transdiagnostic Factors

#### 3.5.1. Avoidance

The first result that emerged from the 23 studies (63.89%) (*n* = 19 experimental studies and *n* = 4 case studies) [39,40,41,42,43,44,45,46,47,48,49,50,51,52,53,54,55,56,57,58,59,60,61] taking into consideration the transdiagnostic factor of “avoidance” was the heterogeneity of the different types of avoidance considered. More specifically, in the majority of the studies (*n* = 13; 56.52%) [41,42,44,45,46,47,50,51,52,54,55,60,61], VR was used to decrease the levels of behavioral avoidance (that is, the individual act of not entering or prematurely leaving a fear-evoking or distressing situation or stimulus) [25] in a wide variety of clinical and non-clinical populations.

Virtual reality exposure treatment (VRET) in particular appeared to be a useful form of intervention to reduce behavioral avoidance in adults with specific phobias (such as flying, driving, going to the dentist, spiders) [41,44,45,54,55,60,61], social anxiety disorder [46], panic disorder with agoraphobia [50,51], and PTSD [52], with results always maintained or even improved [55] over time when a follow-up was present [46,47,51,54,60]. The duration of follow-ups considered spanned between one month and a year. This same technique was also effective in reducing behavioral avoidance in children with a phobia of dogs, with results maintained at a one month follow-up [42]. When compared to other forms of treatment or control conditions, VRET turned out to be more effective than providing informative pamphlets to patients with dental phobia [45] and equally as effective as in-vivo exposure for patients with social anxiety disorder [46] or specific phobias [54]. When traditional forms of psychological therapy were added to VR, such as cognitive therapy [50,51], no additional improvement was found compared to using VR alone. Only in one study were greater changes in behavioral avoidance found in a group of patients with specific phobias (spiders) undergoing one-session treatment (a form of gradated and repeated systematic exposures to the feared stimuli) compared to VRET [55].

Promising results also came from the application of VR-based treatments for the improvement of social avoidance, which is another type of avoidance similar to behavioral avoidance but specific to social situations. This transdiagnostic factor was taken into consideration in six (26.09%) studies, where it was effectively reduced by VR-based treatments in patients with social anxiety disorder [39,46,49,59] and with psychosis [58,59]. Once again, results were maintained over time for both populations (follow-ups range: 3-weeks to 12 months). The combination of VR with cognitive-behavioral therapy (VR-CBT) was also effective in reducing social avoidance more than a waiting list condition in patients with psychosis, with results maintained at six months follow-up [56] and with no differences when compared to traditional CBT in patients with fear of public speaking [59]. Regarding comparisons between VRET and in-vivo exposure, VRET was more effective than the latter in reducing social avoidance in SAD patients and more practical according to therapists in one study [40], but less effective in another [46].

Finally, fewer but promising results were found for the ability of VR-based treatment to improve other types of avoidance, such as agoraphobic avoidance in patients with psychosis [43] or panic disorder with agoraphobia [53], cognitive avoidance in city violence crime victims with PTSD or acute stress disorder [40], alcohol-approach avoidance in patients with substance-use disorder [48], and food avoidance in a patient with bulimia nervosa [57] (see Table S1 for additional details of each study).

#### 3.5.2. Emotion Regulation

Similarly to avoidance, emotion regulation was another transdiagnostic factor that was operationalized in several different ways in the studies. Across the eight articles (*n* = 7 experimental studies and *n* = 1 case study) [62,63,64,65,66,67,68,69] that took into consideration this factor, different dimensions of emotion regulation and emotion regulation strategies were considered.

Three studies [62,66,68] focused on the ability of VR to help regulating emotions by inducing relaxation both in the general population adults [62,66] and adolescents [68]. Interestingly, the characteristics of the VR scenarios seemed to increase this effect. For example, natural VR scenes were shown to increase relaxation more than to control (empty indoor classrooms) VR scenes [62]. The same was seen for VR scenarios where an avatar resembling the participant helped adolescents to regulate their emotions and achieve relaxation more than neutral avatars [68]. VR also produced improvements in relaxation for patients with generalized anxiety disorder when combined with a mindfulness-based intervention [66]. Unfortunately, these studies did not include a control group or a follow-up.

A promising effect of VR in improving emotion expression and regulation was also found in children with autism spectrum disorders undergoing VR training [69], with results better than the waiting list condition although no follow up was included. Healthy adults going through reappraisal-based training in a VR environment also managed to lower the emotional ratings they associated with negative images, showing that VR training can indeed have an impact on emotion regulation. This has been proved also by a case report finding that mindfulness exercises performed in VR reduced negative emotions in a patient with borderline personality disorder and substance use disorder [65].

Moreover, when compared or combined with more traditional forms of psychological interventions, VR also produced some promising results in improving emotion regulation and emotion regulation strategies. The combination of emotion regulation training with risk reduction interventions in VR managed to reduce general population adolescents’ levels of emotional awareness, emotional self-efficacy, emotion regulation strategies and affect regulation, with results often comparable or even better than those obtained by a group using role-playing instead of VR training, and maintained at three-months follow-up [64]. Although VR in combination with a mindfulness-based intervention (MBI + VR) resulted in being as effective as the mindfulness-based intervention alone (MBI) in improving several emotion regulation strategies (i.e., the ability to act with awareness, to control impulses, to self-regulate, to listen to their own body, to describe internal experiences and levels of emotional clarity) in adults with general anxiety disorder [66], MBI + VR even achieved additional improvements in teaching patients to not judge their inner experiences (e.g., thoughts, emotions) and to concentrate even when experiencing negative emotions. Similarly, a VR cognitive-bias modification of interpretations (VR-CBM-I) managed to reduce the emotional response to a stressor more and the resulting sadness more than the standard protocol (CBM-I) [67] (see Table S1 for additional details of each study).

#### 3.5.3. Aggression

Regarding the transdiagnostic factor of aggression, different results were found in three experimental studies carried out in both clinical and general population samples.

The first result showed that VR reduced aggressive behaviors while driving in a sample of war veterans with PTSD, driving anxiety and/or aggression problems, and also helped them to increase their skill training. Even if they rated the virtual experience as not very realistic, the results were maintained at a one-month follow-up [72].

In the second article, using a VR Anger Exposure Training, patients’ levels of anger and aggression after experiencing conflict situations with a friend or a stranger in the virtual environment decreased. In particular, anger scores decreased, especially after anger management exercises when compared to anger expression exercises, regardless if the other person was a stranger or a friend [70].

In the final article, a specific form of VR therapy called Virtual Reality Aggression Prevention Therapy (VRAPT) was able to reduce levels of aggression in forensic patients, even though there was no significant difference with the waiting-list condition. This reduction was also maintained at the three months follow-up [71] (see Table S1 for additional details of each study).

#### 3.5.4. Impulsivity

Impulsivity was a transdiagnostic factor that only produced two articles, out of which one was a multiple case study [73] and one was an experimental study [71], both of which were carried out on clinical populations.

Starting from the experimental study, impulsivity was a secondary outcome taken into consideration in a RCT mainly focusing on aggression, showing results about how this factor decreased after VRAPT treatment was administered on forensic patients. In particular, following treatment, levels of non-planning impulsiveness improved more than in the waiting list condition, with maintenance of results at three months follow-up [71].

In the multiple case study, VR was able to reduce obsessive-compulsive symptoms (such as impulsive thoughts and compulsive behaviors) in three OCD patients, with results maintained at an eight months follow-up [73] (see Table S1 for additional details of each study).

#### 3.5.5. Cognitive Reappraisal

Only two of the articles included in this review [63,74] observed the transdiagnostic factor of cognitive reappraisal (*n* = 1 experimental study and *n* = 1 case study).

The first study was an RCT comparing two different VR training groups (a reappraisal training group and a choice reaction task training group) on their ability to influence the emotional rating participants gave to pictures [63]. Although the main results on the study regarded emotional regulation, this article is relevant in showing that VR technologies can be useful in teaching cognitive reappraisal to healthy adults.

The second study, instead, was a double case study that showed that, following a VR treatment, two children (one with acute anxiety and posttraumatic flashbacks due to past medical treatments and another with suicidal thoughts and low mood) were able to achieve a better expression of their emotions and to reappraise their experience thanks to VR therapy, especially through the perspective-taking feature given by the VR software (ProReal) that was used during treatment [74] (see Table S1 for additional details of each study).

## 4. Discussion

VR has emerged in the literature as a new frontier for the treatment of several psychiatric disorders, with several types of software being developed across the years to tackle a variety of mental disorders [1,2]. However, the disorder-specific approach that has been adopted so far for the development of VR increases the costs needed to apply this technology in the field of clinical psychology [6]. Therefore, the aim of this review was to explore the transdiagnostic potential of VR by searching the literature to investigate the effects of VR-based treatments on a set of six internalizing and externalizing transdiagnostic factors that have been selected for being linked to multiple psychopathologies. Despite these keywords representing only some of the most clinically relevant transdiagnostic factors for mental health, the combination of these keywords with the term “virtual reality” led to a heterogeneous selection of studies carried out on very different clinical and non-clinical populations, further proving the transdiagnostic potential of these factors. While several promising results emerged about the ability of VR to improve these factors in different populations, the review also underlined some differences in the methodological quality of the studies found and in the number of studies carried out on each transdiagnostic factor. Generally, the majority of the results focused on avoidance (especially behavioral and social avoidance) and emotion regulation, while the other transdiagnostic factors (i.e., aggression, impulsivity, cognitive reappraisal and disinhibition) turned out to be understudied. Similarly, patients with anxiety disorders represented the most studied clinical population, but interesting applications of VR on other clinical and non-clinical populations (e.g., PTSD, psychotic disorders, eating disorders, OCD, etc.) were also found. Moreover, although more than half of the studies were controlled (with a prevalence of RCTs and a few non-randomized controlled trials), only a small proportion compared VR-based interventions with traditional psychotherapies (e.g., CBT, Mindfulness, DBT, etc.) and a waiting-list control condition was often preferred. The absence of studies using VR in combination with drug therapy to improve these transdiagnostic factors also did not make it possible to collect information about the potential of VR to improve the results of pharmacotherapy or promote adherence to drug therapy for different mental health patients. Follow-ups were also included in more than half of the studies, but maintenance of results over time was never investigated beyond one year. These differences in methodologies across the articles explains the fact that only half of them reached a strong quality score.

More specifically, the transdiagnostic factors that were the focus of most of the selected studies were behavioral and social avoidance, which are largely associated with anxiety disorders [75,76,77]. This was not surprising, considering that the very first applications of VR in clinical psychology consisted in using this technology to provide an alternative to in-vivo exposure [78,79]. In particular, through the use of VR, patients can improve their conditions by being exposed virtually to situations or objects that elicit the same sense of discomfort as the ones in real life [79,80], thus reducing avoidance of these stimuli.

The results of the review further underline how this is still the way VR is most frequently implemented in psychological treatments, with very positive results. Indeed, in all the studies found, VR was able to reduce behavioral and social avoidance between pre- and post-treatment, confirming how this technology may represent a promising psychological tool that was also as effective as traditional in vivo exposure in some studies [39,46,54]. However, various studies compared VR or used VR in combination with first-line treatments tackling behavioral and social avoidance, such as CBT. While these studies showed that VR combined with cognitive-behavioral therapy (VR-CBT) was effective in reducing social avoidance long-term [56] and with no differences when compared to traditional CBT [59], further studies are definitely needed in order to confirm these promising results.

Similarly, while VR was found to improve other types of avoidance (e.g., agoraphobic avoidance, cognitive avoidance, food avoidance, and alcohol-approach avoidance) that are linked to disorders outside of the anxiety category (e.g., psychosis, PTSD, substance-use disorders, and eating disorders, respectively) [40,43,48,53,57], our review of the literature was not able to find more than a few studies for these factors and clinical populations. Indeed, since the literature showed that different kinds of avoidance can be involved in the maintenance or development of different kinds of pathologies [81,82,83], investing in a VR software capable of tackling this factor transdiagnostically would be of clinical relevance.

Another transdiagnostic factor that plays a role in several psychopathologies is emotion regulation, which was also the second most found factor in the review. VR interventions were capable of increasing relaxation and diminishing negative emotions, particularly fear and anger, in several studies carried out in the general population [62,65,68], as well as of teaching emotion regulation strategies in clinical and non-clinical participants [63,66,69]. Also successful was the combination of VR with other traditional interventions meant to improve emotion regulation, such as Mindfulness-Based Interventions [65,66]. However, once again, studies comparing or combining VR with other forms of interventions were few, and further research is needed to test these results. The ability of VR to improve emotion regulation would hold clinical utility not only for the treatment of psychological disorders, but also for their prevention, as several authors underline how difficulties in emotion regulation are strictly linked to the development of several psychopathologies (e.g., anxiety and mood disorders, eating disorders, substance-related disorders, and more) [84,85]. Moreover, many of the types of software that tackle emotion regulation can already be considered transdiagnostic in nature because they can be used in many different populations, although there are no studies about the same software being applied to improve this transdiagnostic factor across clinical samples with different psychiatric diagnoses.

On the other hand, not many studies appeared for the transdiagnostic factors of impulsivity, aggression, and cognitive reappraisal. Nonetheless, the up-to-date literature showed promising results about VR software lowering levels of impulsivity and impulsive behaviors in specific populations, more specifically patients with OCD [73] and in forensic patients [71]. In this latter population, VR was also capable of lowering levels of aggression [71] through Virtual Reality Aggression Prevention Therapy. By using VR, aggressive behaviors were also reduced in veterans with PTSD when driving [72] and in people of the general population [70], further proving that a single transdiagnostic VR software would have the potential to be applied on multiple clinical and non-clinical populations.

Finally, VR also emerged as a tool to teach cognitive reappraisal in children, highlighting the intergenerational potential of VR interventions to help clinicians to create a better alliance with children during treatment [74]. VR cognitive reappraisal training could also be used alongside emotion regulation training in VR, since it has been seen that participants rated negative images less severely after undergoing this kind of virtual training [63]. Tackling more than one transdiagnostic factor with a single piece of software would further decrease the costs linked to VR technologies. This would lead to a more frequent implementation of VR in the clinical field, which in turn might help to engage more people towards seeking psychological treatment, especially treatments that target cognitive reappraisal, such as CBT. Indeed, the resemblance between VR and technology used in everyday life could help to lower the stigma associated with traditional psychotherapy.

## 5. Conclusions

Results of this review further supported the use of VR in clinical psychology, in particular for improving transdiagnostic factors. Moreover, VR has also shown similar results compared to CBT, especially when treating behavioral avoidance [40,51,59], which suggests that it might be a valid alternative to traditional psychotherapies for anxiety disorders. Third-wave cognitive-behavioral therapies, such as mindfulness-based intervention, might also benefit from the addition of a technological VR tool [65,66]. However, additional studies are needed to prove the transdiagnostic potential of VR (in particular or what concerns its ability to improve aggression, impulsivity, and cognitive reappraisal, as well as understudied forms of avoidance), and clinicians still need to work on developing VR software that are truly transdiagnostic in nature and on testing them on more varied clinical and non-clinical populations. Moreover, while promising, the results of the present review need to be considered in light of its methodological limitations.

The main limitation of this review was the choice of a limited and arbitrary number of keywords representing transdiagnostic factors for the literature search. Indeed, we were not able to find a shared, set list of transdiagnostic factors to consider for our keywords. Although an attempt was made to choose the main internalizing and externalizing factors for mental disorders that are also linked to a wide number of psychopathologies [10,11,12,15,26,27,28,29,30,31,32,33,34,35,36], this arbitrary selection might also have led to the neglect other important keywords. Future reviews might help with investigating the effects of VR-based interventions on other important transdiagnostic factors. Similarly, conducting the bibliographic research using only two databases (PubMed and PsycINFO) and only choosing articles published after 2010 might have led to the exclusion of other relevant studies. While not mandatory, not registering the systematic review protocol on any public repository (e.g., PROSPERO) might be another limitation of the review. However, although a PROSPERO registration has become a widely recommended practice for systematic reviews over the past few years, no differences in quality of research has been found in the literature between registered and non-registered systematic reviews [86], and the use of PRISMA and PICOS criteria encourages and allows replicability of results.

Other limitations were also related to the quality and methodologies of the studies found. Indeed, future research should focus on carrying out studies with a stronger quality and less risk of bias, especially by including follow-ups and RCTs comparing VR to other more traditional psychotherapies (e.g., CBT, mindfulness, DBT, etc.), at least for what concerns the effects of VR-based interventions on transdiagnostic factors. Future studies may also try to investigate the transdiagnostic potential of VR when used in combination with drug therapy and whether VR may help with increasing adherence to treatment.

Ultimately, since the great majority of the articles had adult samples (with only very few studies carried out on adolescents or children), future studies should also consider testing the application of VR-based interventions for the improvement of transdiagnostic factors in people of different ages. Similarly, VR research on transdiagnostic factors should try to expand more outside the field of anxiety disorders and to explore the use of VR in other understudied clinical and non-clinical populations, including the general population at risk for the development of psychopathologies.

## Figures and Tables

**Table 1:** Population, Intervention, Comparison, Outcomes and Study (PICOS) table for inclusion and exclusion criteria [37].

PICOS	Inclusion Criteria	Exclusion Criteria
Patients	Patients with mental health disorders Individuals from the general population	Individuals with physical diseases, hospitalized due to physical conditions or presenting brain damage
Intervention	Any intervention that included the use of Virtual Reality technologies	Interventions that did not include the use of Virtual Reality Neuroscience studies
Control Group	Any control group or absence of a control group	None
Outcome	Any type of change in patients’ levels of emotion regulation, avoidance, impulsivity, aggression, reappraisal, and disinhibition.	None
Study Design	Longitudinal studies (pre-post intervention) Randomized and non-randomized controlled trials Case studies	Study protocols Systematic and non-systematic reviews Meta-analyses Medical or neuroscience studies Books (or book chapters)

## Supplementary Materials

The following supporting information can be downloaded at: https://www.mdpi.com/article/10.3390/jcm11216463/s1, Table S1: Characteristics, transdiagnostic factors considered and relevant outcomes of selected studies; Table S2: Quality assessment and risk of bias criteria for observational cohort, case–control, and controlled intervention studies; Table S3: Assessment of quality and risk of bias of selected experimental studies.
