# Supplementary material for: The Effects of Virtual Reality in Targeting Transdiagnostic Factors for Mental Health: A Systematic Review of the Literature
Source: J Clin Med. 2022 Oct 31;11(21):6463. doi: 10.3390/jcm11216463 (PMC9656930; doi:10.3390/jcm11216463)
Supplement: Supplementary file 1 [file jcm-11-06463-s001.zip › jcm-1960212-supplementary.pdf]

Supplementary materials

Supplementary Table S1. Characteristics, transdiagnostic factors considered and relevant outcomes of selected studies.

Author	Age (M±SD, or range when mean not available)	Gender	Sample type (n)	Transdiagnostic factor	Study Design	Follow-up	Measurement tool for considered transdiagnostic factors	VR technology type (software)	Relevant results
**Avoidance**									
Bouchard et al., 2017 [39]	CBT + in virtuo exposure (36.2±14.99); CBT + in vivo exposure (36.7±11.1); WL (30.6±9.1)	43 females, 16 males<br><br>CBT + in virtuo exposure (15 females, 2 males); CBT + in vivo exposure (17 females, 5 males); WL (11 females, 9 males)	Adults (59) with a diagnosis of SAD for at least the past 2 years.	Avoidance (Social Avoidance)	Randomized Controlled Trial comparing: CBT + in virtuo exposure (n=17) vs CBT + in vivo exposure (n=22) vs WL (n=20)	6-months	LSAS-SR	Immersive VR (Virtually Better)	VRE was considered more practical than IVE by therapists according to SWEAT scores (CBT+VRE: 15.24±3.96; CBT+IVE: 24.46±9.85; $t_{(22.83)} = 3.66$, $p<.001$). VRE was better than IVE in reducing social anxiety and avoidance (LSAS-SR; CBT+VR: Pre = 85.1±29.5, post = 51.8±23.3; CBT +IVE: pre = 74.9±24.5, post = 56.0±26.9, p < 0,05) and social phobia (SPS; CBT + VR: pre = 39.0±16.1, post = 19.2±12.5; CBT + IVE: pre = 30.9±17.5, post = 22.4±15.7, p < 0.001).
Cárdenas-López et al., 2014 [40]	Females (36.5±14.4); Males (34.7±16.39)	2 females; 7 males	City violence crime victims (9) undergoing VRET for PTSD (n=6) or VRET for ASD (n=3)	Avoidance (Cognitive Avoidance)	Uncontrolled clinical trial	No	CAPS-Avoidance	Immersive VR	All the participants undergoing VRET showed an improvement on PTSD symptoms after the 12 week program, including avoidance (CAPS Avoidance: pre 32.2±13.2; post 2.3±1.8, p<0.001).
Czerniak et al., 2016 [41]	26, 50 and 51 years old	3 males	Men suffering from panic attacks, phobias and fear of flying (n=3)	Avoidance (Behavioral Avoidance)	Multiple case studies	No	//	Immersive VR (CAREN)	After VRET 1 patient stopped taking anxiety medication before boarding a plane, the second patients stated that almost two years after treatment had flown several times, finally the third patient did not fly after treatment

Farrell et al., 2020 [42]	10.25±2.11	4 females, 4 males	Children with specific phobias (dogs) (8)	Avoidance (Behavioral Avoidance)	Multiple case studies	1-month	BAT	Immersive VR	Behavioural avoidance lowered from pre to post-treatment (Z = -2.21, p = .02) and from pre-treatment and a month follow up (Z = -2.38, p = .008). Post-treatment results were maintained at follow-up (p = .50).
Freeman et al., 2022 [43]	gameChange VR therapy + usual care (36.6±12.8); usual care alone (37.8±12.2)	111 females, 231 males<br><br>gameChange VR therapy + usual care (58 females, 116 males); usual care alone (53 females, 115 males)	Adults with psychosis or affective disorder diagnosis with psychotic symptoms presenting agoraphobic symptoms (346)	Avoidance (Agoraphobic Avoidance)	Multicentre, parallel-group, single-blind randomized controlled trial comparing: gameChange VR therapy + usual care (n=174) vs usual care alone (n=172)	6 weeks	O-AS; O-BAT	Immersive VR (gameChange)	Compared with the usual care alone group, the VR group significantly reduced agoraphobic avoidance (O-AS adjusted mean difference = –0.47, p=.026; O-BAT adjusted mean difference = 0.89, p=.0004) at 6 weeks.
Gujjar et al., 2018 [44]	n.a.	n.a.	Adults with dental phobia (10)	Avoidance (Behavioral Avoidance)	Uncontrolled clinical trial	6-months (but not for BAT scores)	BAT	Immersive VR	VRET was able to decrease the behavioral avoidance between pre and post-intervention (BAT: pre = 41.2±5.5; post = 12.8±7.5, *d* = 4.2, p < 0.05)
Gujjar et al., 2019 [45]	VRET (25.3±8.6); IP (23±8.9)	18 males, 12 females<br><br>VRET (8 females, 7 males); IP (10 females, 5 males)	Adults with dental phobia (30)	Avoidance (Behavioral Avoidance)	Randomized controlled trial comparing VRET (n=15) vs IP (n=15) group	6-months (but not for BAT scores)	BAT	Immersive VR	VRET improved behavioral avoidance between pre and post-treatment (BAT: pre=38.4±4.5, post=21.8±8.0, $t_{(14)}$ = 8.09, p < 0.01; BAT steps: pre=2.1±0.3, post=3.5±0.6, $t_{(14)}$ = −7.36, p < 0.001), with better results when compared to the IP group at post-treatment (BAT: VRET=38.4±4.5, IP=33.4±2.8, *d* = 1.33, p<.05; BAT steps: VRET=21.8±8.0, IP=32.7±2.2, *d* = 1.86, p<.05)
Kampmann et al., 2016 [46]	VRET (39.65±11.77); iVET (37.50±11.27); WL (33.50±11.44)	VRET group (65% females); iVET group (75% females); WL (50% females)	Adults with SAD (60).	Avoidance (Social Avoidance, Behaviora	Randomized controlled trial comparing: VRET (n=20) vs iVET (n=20) vs WL (n=20)	3-months	LSAS-SR	Immersive VR	Social avoidance and anxiety was reduced between pre and post treatment in both the iVET (LSAS-SR: pre=69.15±19.44, post=39.22±25.01, p < .001, d=1.14) and the VRET group (LSAS-SR: pre=73±17.25, post=55.74±18.65, p=.014; d=0.55), with a greater decrease in iVET

				l Avoidance)					(p=.006). No significant differences were found between VRET and the waiting-list control group between pre and post-treatment (p=.197). Social avoidance slightly increased at 3-month follow-up for the VRET group (LSAS-SR: 57.89±23.60, d=0.55). Behavioural avoidance improved for both VRET (p=.018; *d* = 0.56) and iVET (p = .002; *d* = 0.77) compared to the WL. No significant differences were found between VRET and iVET at postassessment (p=.920)
Kaussner et al., 2020 [47]	40.36±8.57	5 males and 9 females	Adults with fear of driving (n = 14).	Avoidance (Behavioral Avoidance)	Non-Randomized Controlled Study comparing: VRET (n=9) vs WL (n=4)	12-weeks	BAT	Non-immersive VR (SILAB)	VRET was useful to reduce avoidance and fear of driving, with all the participants completing one task that they avoided previously (Friedman test X^2 = 14.85; p = .002). 93% of patients maintained the results at follow-up.
Kim & Lee, 2019 [48]	VAAAT (22.36±2.31); Control/sham training Group (22.79±2.89)	VAAAT Group (8 males, 6 females); Control/sham training Group (9 males; 5 females)	Heavy social drinkers (28)	Avoidance (Alcohol-Approach Avoidance)	Randomized Trial comparing: VAAAT (n=14) vs Control/sham training group (n=14)	No	A-IAT	Immersive VR (VAAAT)	In the VAAAT ($t_{(13)}$ = 2.77, p < 0.05, *d* = 0.42), alcohol-approach avoidance increased, while it decreased in the control group ($t_{(13)}$=-4.38, p<0.01, *d*=0.78). However, the main effect of group [$F_{(1,26)}$ = 0.01, p = 0.92] and time [$F_{(1, 26)}$ = 1.05, p = 0.32] was not statistically significant.
Kim et al., 2020 [49]	n.a. (range: 19-30 years)	n.a.	Adults with SAD (52)	Avoidance (Social Avoidance)	Randomized Controlled Trial comparing: VR (n=24) vs WL (n=28)	3-weeks	LSAS	Immersive VR	After VR treatment, LSAS total ($F_{(1,39)}$ = 5.8, p = 0.02), anxiety ($F_{(1,39)}$ = 6.9, p = 0.01) and social avoidance decreased ($F_{(1,39)}$ = 4.7, p = 0.04). At follow-up anxiety and social avoidance lowered significantly (t_{20} = −3.8, p < 0.01; and t_{20} = −3.2, p < 0.01, respectively). In the WL group, no significant changes in these scores were found.
Malbos, Rapee & Kavakli, 2011 [50]	n.a.	8 females and 2 males	Adults with panic disorder and agoraphobia (10)	Avoidance (Behavioral Avoidance)	Randomized Controlled Trial comparing: VRET (n=n.a.) vs VRET + cognitive therapy (n=n.a.)	No	BAT	Immersive VR	Behavioral avoidance decreased in the VRET only group (BAT: pre=8.20±2.4; post=9.6±0.89, p <0.025) with no significant difference with the VRET + cognitive therapy group (BAT: pre=5.0±3.19, post=7.8±2.68, p <0.025).
Malbos, Rapee &	44.11±13.79	12 females and 7 males	Adults with panic disorder	Avoidance	Randomized Controlled Trial	3-months	BAT	Immersive VR	Beavioral avoidance decreased both in the VRET only group (BAT: pre=9.0±2.0, post=9.78±0.67,

Kavakli, 2013 [51]			and agoraphobia (19).	(Behavioral Avoidance)	comparing: VRET (n=n.a.) vs VRET + cognitive therapy (n=n.a.)				follow-up= 9.87±0.35, $p < 0.025$) and in the VRET + cognitive therapy group (BAT: pre=7.22±3.19, post=8.78±2.22, follow-up=8.62±2.32, $p < 0.025$), with maintenance of results at 3-months follow-up. No significant difference was found when adding cognitive therapy.
Maples-Keller et al., 2017 [52]	34.74±8.35	141 males, 9 females	Iraq and/or Afghanistan veterans with PTSD (150)	Avoidance (Behavioral Avoidance)	Uncontrolled clinical trial (secondary analyses from a randomized controlled trial)	No	PSS	Immersive VR	According to three different measurements in different period of time, VR reduced behavioural avoidance symptoms between pre and post-intervention in the sample (t1 = 4.25±1.65); t2 = 3.56±2.07); t3 = 2.98±2.11); *d* = .77)
Meyerbroeker et al., 2013 [53]	n.a. (range: 18-65 years)	n.a.	Adults with panic disorder with agoraphobia (55).	Avoidance (Agoraphobic Avoidance)	Randomized controlled trial comparing: VRET (n=19) vs iVET (n=18) vs WL (n=18)	No	MI	CAVE,	No difference was found between VRET and iVET in improving agoraphobic avoidance (MI: $F_{(2, 27)} = 0.882$, $p = 0.425$) and active treatments produced greater changes than WL ($F_{(1, 33)} = 15.181$, $p = 0.000$).
Michaliszyn et al., 2010 [54]	29.1±7.99	31 females and 1 male	Adults with specific phobia (spiders) (32).	Avoidance (Behavioral avoidance)	Randomized controlled clinical trial comparing: VRET (n=n.a.) vs iVET (n=n.a.)	3-months	BAT, FSQ-F	Immersive VR	VRET produced a reduction in behavioral avoidance between pre and post-treatment (BAT: pre= 3.56±2.89; post=9.25±2.72, $p<.001$), with results maintained at follow-up (BAT=9.73±2.43). No significant difference was found between VRET and iVET for this variable ($F_{(1, 24)} = 2.55$, $p = 0.12$). Both iVET (FSQ-F: pre=103.28±13.13; post=47.88±14.07; follow-up=47.81±32.25) and VRET (FSQ-F: pre=104.61±9.59; post=54.37±22.46; follow-up=56.67±23.99) improved fear and avoidance of spiders at post-treatment and follow-up (time effect: $F_{(6, 28)}=17.12$, $p= 0.00$), with no difference between treatments ($F_{(1,24)}=0.814$, $p=0.445$).
Miloff et al., 2019 [55]	OST group (34.04±9.85); VRET (34.06±10.92)	OST (8 males, 41 females and 1 other); VRET (8	Adults with specific	Avoidance (Behavioral	Randomized controlled trial comparing: one session treatment	12-months	BAT	Immersive VR	Behavioural avoidance improved at post-treatment in both OST (BAT: pre=5.66±2.47, post=10.70±1.68, $\beta = 4.84$, 95% CI 4.15 to 5.52, $d = 2.39$) and VRET (BAT: pre=4.76±2.71,

		males, 42 females)	phobia (spiders) (100).	Avoidance)	(n=50) vs VRET (n=50)				post=8.50±2.29, β = 3.55, 95% CI 2.87 to 4.23, d = 1.49) groups, but with significantly greater improvements in OST (β = −1.27, 95% CI -2.27 to −0.28, p = .013). The VRET group continued to experience significant improvements between post-treatment and 12-months follow-up (BAT=9.36±1.77) compared to the OST group (BAT=10.58±1.81) (β = 0.38, 95% CI .13 to .63, p = .002).
Pot-Kolder et al., 2018 [56]	VR-CBT (36.5±10); WL group (39.5±10)	VR-CBT (40 males, 18 females); WL (42 males, 16 females)	Adults with psychotic disorders (116).	Avoidance (Social Avoidance)	Single-blind randomized controlled trial comparing: VR-CBT (n=58) vs WL (n=58)	6-months	ESM; SBQ	Immersive VR (Vizard)	Time spent with others did not increase between pre and post-treatment in the VR-CBT group (ESM: pre=0.416±0.26; post=0.404±0.24; p=.178), but it increased between pre-treatment and follow-up (ESM: follow-up: 0.419±0.24, p = 0.009). Time spent with others did not increase in the WL group (ESM: pre=0.364±0.27, post=0.323±0.28, follow-up= 0.340±0.30). Compared with the WL group (SBQ: pre=24.1±15, post=23.8±16.5, follow-up=22.5±13.5), use of safety behaviours decreased significantly in the VR-CBT (SBQ: pre=28.8±14.2, post=21.1±16, follow-up=20.2±16.2) group at both post-treatment and follow-up assessment (b interaction=-3.7, z=–2.93, p=0·0033).
Roncero & Perpiñá, 2015 [57]	22 years old	1 female	Adult with bulimia nervosa (1)	Avoidance (Food Avoidance)	Case study	No	Ad-hoc intra-session questionnaire	Non-immersive VR	VR had positive effects on eating patterns for the patients, especially for food avoidance from session 1 (10) to session 7 (6) with a complete absence of this symptom (0) at session 6.
Rus-Calafell et al., 2014 [58]	36.50±6.01	7 males and 5 females	Adults with schizophrenia or schizoaffective disorder (12)	Avoidance (Social Avoidance)	Uncontrolled clinical trial	4-months	SADS	Immersive VR (Soskitrain program)	Social avoidance decreased between pre (SADS=7.75±0.81) and post VR treatment (SADS=4.08±0.41) (p=.001), maintaining the result at follow-up (SADS=4.17±0.53, p < 0.05).
Safir, Wallach	27±n.a.	68 females and 20 males	PSA patients (88).	Avoidance	Follow-up of a Randomized	12-months follow-up	LSAS	Immersive VR	At follow-up there were no significant differences between CBT (LSAS=15.50±15.43)

& Bar-Zvi, 2012 [59]				(Social Avoidance)	Clinical Trial comparing: VR-CBT (n=28) vs CBT (n=30) vs WL (=30)	of Wallach, Safir & Bar-Zvi (2009)		(Virtually Better)	and VR-CBT (LSAS=14.36±11.37) for avoidance ($F_{(1, 46)}$ = 0.45, p = .50). VR-CBT maintained results at follow-up ($t_{(47)}$ = 1.52, p = .14).
Shiban et al., 2015 [60]	31.14±10.78	n.a.	Adults with specific phobia (spiders) (32).	Avoidance (Behavioral Avoidance)	Randomized Controlled Clinical Trial comparing: VRET/reactivation group (n=15) vs VRET/no reactivation group (n=17)	6-months	BAT; 7-item ad-hoc follow-up questionnaire	Immersive VR (Cybersession)	Behavioral avoidance decreased in both groups, as it was shown by the time effect ($F_{(1, 25)}$ = 11.38, p < .01). No significant difference was found between groups (group*time interaction: $F_{(1, 25)}$=.39, p = .54). At the 6-months follow-up both groups reported low levels of avoidance in contact with spiders (VRET/reactivation group: ad-hoc questionnaire=3.18±2.92; VRET/no reactivation group: ad-hoc questionnaire =3.57±2.31), with no significant difference between groups (p = 0.71).
Wrzesien et al., 2015 [61]	41.50±17.52	4 females	Adults with specific phobias (spiders) (4)	Avoidance (Behavioral Avoidance)	Multiple single-case studies	3 and 12-months	BAT	Immersive VR (P-ARET)	Behavioral avoidance decreased between pre treatment (BAT=7.75±1.50) and post treatment (BAT=4.50±3.70) (Z = -1.826; p = .068), between pre-treatment and 3 months follow-up (BAT=5.75±2.22) (Z = -1.826; p = .068), and between pre-treatment and 12 months follow-up (BAT=1.75±2.87) (Z = -1.826; p = .068).
**Emotion Regulation**									
Anderson et al., 2017 [62]	32±12	9 males and 9 females	General Population (18)	Emotion Regulation	Uncontrolled clinical trial	No	Physiological variables (HRV, LF/HF)	Immersive VR	Natural VR scenes increased relaxation over other control VR scenes, decreasing stress from beginning (LF/HF: control scene=2.00±1.86; dream beach scene=1.00±0.63; Ireland scene=2.27±1.83) to end (LF/HF: control scene=1.38±1.28, p = < 0.05; dream beach scene= 0.92±0.36, p< 0.05; Ireland scene=1.88±1.75, p< 0.05).
Bosse et al., 2014 [63]	28.2±n.a. (range: 26-32 years)	6 females and 9 males	General population/healthy adults (15)	Emotion Regulation;	Randomized Controlled Trial comparing: choice reaction task (n=5)	6-months	PLUX wireless sensor device	Non-Immersive VR (IAPS)	In the reappraisal group, emotional rating lowered for all images [$t_{(298)}$ = 4.342, p < 0.0001] as well as for the negative pictures only [$t_{(113)}$ =

				Reappraisal	vs reappraisal group (n=5) vs control/no training group (n=5)				1.7808, p = 0.039] post VR task, with results maintained at follow-up.
Hadley et al., 2019 [64]	ER + RP (13±0.91); ER + IVRE (12.9±0.82)	ER + RP (54% females, 46% males); ER + IVRE (55% females, 45% males)	General population adolescents (88)	Emotion Regulation	Randomized Controlled Trial comparing: ER + RP (n=42) vs ER + IVRE (n=46)	3-months	ADS; DERS	Immersive VR	Difficulty in Emotional Awareness decreased in both groups between baseline and the 3 months assessment (ER + IVRE: *d*=-0.50, p<.05; ER+RP: *d*= -0.61, p<.01) with no difference between conditions (*d* =0.09, p = .67). Emotional self-efficacy at the 3-month assessment increased more in the ER+IVRE group (*d* = 0.26; p =.23) while ER + RP participants only reported minimal improvement (*d* = 0.00, p =.91) with a small difference between conditions (*d* =0.20, p = .36). Difficulty Accessing Emotion Regulation Strategies did not change change in the ER+IVRE group and the 3 months assessment but increased in the ER+RP group (*d* =0.71, p < .01). The ER+ IVRE group noted a small increase in affect dysregulation (*d* = 0.21, p = .35), while the ER + RP condition reported a small-to-moderate increase in dysregulation at 3 months (*d* = 0.36, p = .10) with minimal difference between conditions (*d* = 0.13, p = .59).
Navarro-Haro et al., 2016 [65]	32 years old	1 female	Patient with BPD and SUD (1)	Emotion Regulation	Single case study	No	DBT diary card; KIMS-Short	Immersive VR (MindfulRiver World)	Three VR mindfulness exercises were useful in reducing negative emotions in the patient from session 1 (fear=20 vs 0; anger= 0 vs 0; guilt=40 vs 10; disgust= 40 vs 0 and joy=10 vs 30) to session 2 (fear=30 vs 10; anger=20 vs 0; guilt=20 vs 0; shame=20 vs 10; disgust=20 vs 0 and joy=30 vs 30).
Navarro-Haro et al., 2019 [66]	45.23±11.23<br><br>MBI group (45.40±13.74); MBI + VR	30 females, 9 males	GAD patients (39).	Emotion Regulation	Randomized Controlled Trial comparing: MBI (n=20) vs MBI+VR (n=19)	No	VAS; MAIA; DERS; FFMQ	Immersive VR (MindfulRiver World)	MBI+VR achieved significant pre-post improvements in FFMQ-describing internal experiences (p=.001), FFMQ-acting with awareness (p=.003), DERS-emotional clarity (p=.014), DERS-impulse control (p=.001),

	group (45.05±8.17).	MBI group (15 females, 5 males); MBI + VR group (15 females, 4 males)							MAIA-self-regulation (p<.001), MAIA-listening to body (p=.016), with no difference when compared to the MBI group. The MBI+VR group exhibited additional improvements in FFMQ-non-judging inner experiences (p = 0.024); and DERS-difficulties concentrating when experiencing negative emotions (p < 0.001). The MBI+VR group also showed improvements of relaxation in all VR sessions.
Otkhmezuri et al., 2019 [67]	21.60±2.96 VR-CBM-I (21.05±1.91); standard CBM-I (22.14±3.7)	23 females, 19 males. VR-CBM-I (14 females, 7 males); standard CBM-I (9 females,12 males)	General population students (42).	Emotion regulation	Non-randomized controlled study comparing: CBM-I (n=21) vs VR-CBM-I (n=21)	No	VAS to evaluate emotional responses to a stressor (anxiety and sadness)	Immersive VR	The VR-CBM-I group reported a greater reduction in the emotional response to the stressor (assessed by the VAS Anxiety) compared to the CBM-I group with a significant group ($F_{(1,40)}$=15.4, p<.001) and time*group ($F_{(1,40)}$=5.2, p=.03) effect. In particular, the stress task resulted in a significant increase in anxiety in the CBM-I group (t_{20}=−3.3, p=.003, *d*=0.72), but not in the VR-CBM-I training (t_{20}=−1.4, p=.18, *d*=0.31). The VR-CBM-I also reported less levels of sadness after the stress task than the CBM-I group (group effect: $F_{(1,40)}$=12.2, p=.001), but the time*group effect was not significant ($F_{(1,40)}$=2.7, p=.09).
Wrzesien et al., 2015 [68]	13.27±0.47 VRN group (13.27±0.47); VRS group (13.17±0.39)	11 males, 11 females	General population adolescents (22)	Emotion Regulation	Randomized controlled trial comparing: VRS vs VRN group	No	VAS; SAM	Immersive VR	VAS scores showed that both group experienced an increase in relaxation following the emotion regulation phase of the test ($F_{(2, 42)}$ = 3.957, p = 0.027), with no significant group ($F_{(1, 21)}$ = 1.710, p = 0.20) or phase*group effect ($F_{(2, 42)}$ = 0.793, p = 0.45). However, pairwise comparisons showed that relaxation increased significantly after the regulation phase in the VRS group especially (p=.011). SAM showed that calm increased in both groups between baseline and after the regulation phase (p = 0.026 for both groups). There was also a significant decrease in the arousal dimension

									after the regulation phase in the VRS group (p = 0.011).
Yuan & Ip, 2018 [69]	106.3±13.5 months<br><br>VR training group (107.6±13.27 months); WL (104.8±13.83 months).	64 males, 8 females<br><br>VR training group (31 males, 5 females); WL (33 males, 3 females)	Children with autism spectrum disorders (72)	Emotion Regulation	Non-randomized controlled study comparing: VR training (n=36) vs WL (n=36)	No	PEP-3	CAVE	Children that underwent VR training obtained an improvement in emotion expression and regulation (pre=18.9±3.57, post=20.2±3.00, p = .037) and in social interaction and adaptation (pre=20.2±3.43, post=21.8±2.99, p < .0005). Finally, a statistically significant interaction between group and time on affective expressions ($F_{(1, 70)}$ = 5.223, p = .025, *partial* η^2 = .069) and on social reciprocity ($F_{(1, 70)}$ = 7.769, p = .007, *partial* η^2 = .100) was found in favour of the VR training group.
**Impulsivity**									
Laforest et al., 2016 [73]	n.a. (2 adults in their mid-20s, 1 adult in her mid-30s)	3 females	Adults with OCD (3)	Impulsivity	Case studies	8-months	Self-monitoring of obsessions and compulsions; YBOCS	CAVE	VR intervention improved OCD symptoms (impulsive thoughts and compulsive behaviours) for all three participants, as it can be seen by the YBOCS results (patient 1: pre =22, post = 14, 4 months f-u = 16, 8 months f-u = 21; patient 2: pre = 31, post = 14, 4 months f-u = 11, 8 months f-u =11; patient 3 pre = 30, post = 21, 4 months f-u = 23, 8 months f-u = 27).
**Cognitive Reappraisal**									
Falconer et al., 2019 [74]	12 and 15 years old	2 males	1 participant with acute anxiety and posttraumatic flashbacks due to medical treatments; 1 participant with suicidal thoughts and low mood.	Reappraisal	Case studies	No	n.a.	Non-Immersive (ProReal)	Both participants were able to better express their emotions, reappraise their experience, and learn perspective taking through the VR visualization.
**Aggression**									
Jo et al., 2022 [70]	High Aggression	60 males	General population with	Aggression	Non-Randomized Controlled Trial	No	STAXI	Immersive VR	VR Anger Exposure Training was effective in teaching participants with low and high levels of

	Group (23.9±2.5); Low Aggression group (24.1±2.6)		high and low levels of aggression (60)		comparing: people with high (n=30) and low levels of aggression (n=30)				aggression to manage anger expression. In particular, participants' levels of anger decreased after managed expression both when in the VR scenario "conflict with a friend" (high aggression group: $t_{(29)} = 8.78$, $p < 0.001$; low aggression group: $t_{(29)} = 9.90$, $p < 0.001$) and in the VR scenario "conflict with a stranger" (high aggression group: $t_{(29)} = 9.90$, $p < 0.001$; low aggression group: $t_{(29)} = 11.11$, $p < 0.001$) with no significant difference between groups.
Klein Tuente et al., 2020 [71]	VRAPT (39.4±10.6) WL (38.0±10.0)	n.a.	Forensic patients (128)	Aggression; Impulsivity	Multicentered randomized controlled trial comparing: VRAPT (n=58) vs WL (n=64)	3 months follow-up	SDAS; AVL; BIS-11; BDHI-D; NAS-PI; STAXI-2; RPQ	Immersive VR	Self-reported aggression decreased both in the VRAPT and WL group between pre and post-treatment ($F_{(1.91)}$=101.04, p = 0.17) and between pre-treatment and 3 months follow-up ($F_{(1.44)}$=100.42, p = 0.23), but there was no difference between groups. Staff-rated aggression did not change significantly between pre and post-treatment in any of the groups. VRAPT obtained better results than WL in aggression and hostility (BDHI-D total; p=.02), direct aggression (BDHI-D subscale; p=.04), non-planning impulsiveness (BIS-11 subscale; p=.04), anger control (STAXI-2 subscale; p=.02), and anger expression (STAXI-2 subscale; p=.03), but no treatment effect was found at 3-months follow-up.
Zinzow et al., 2018 [72]	n.a.	8 males	Veterans with PTSD, driving anxiety and/or aggression problems (8)	Aggression	Uncontrolled clinical trial	1-month	DBS	Non-Immersive (DriveSafety CDS-250)	Even though participants did not feel the simulation as realistic, VRET+CBT lowered their levels of hostile/aggressive behavior while driving between pre and post-treatment (DBS subscale: pre= 3.71±1.18, post = 1.81±0.99, p <0.01), with the maintenance of results at 1-month follow-up (=1.64±0.62).

Abbreviations. ADS = Affect and Disregulation Scale; AFQ-Y = Avoidance and Fusion Questionnaire for Youth; A-IAT = The Alcohol Approach-Avoidance Implicit Association Test; ASD = Acute Stress Disorder; AVL = Aggression Questionnaire; BAT = Behavioral Assessment Task; BDHI-D = Buss-Durkee Hostility Inventory-Ductch; BIS-11 = Barratt Impulsivness Scale; BPD = Borderline Personality Disorder; CAPS = Clinician Administered PTSD Scale for DSM-IV; CAREN = Computer Assisted

Rehabilitation Environment; CAVE= Cave Automatic Virtual Environment; CBM-I = Cognitive Bias Modification of Interpretations; CBT = Cognitive Behavioral Therapy; DBS = Driving Behavior Survey; DBT = Dialectical Behavioral Therapy; DERS = Difficulties in Emotion Regulation Scale; ER = Emotion Regulation; ESM = The experience sampling method; FFMQ = Five facets of mindfulness questionnaire; FSQ = Fear of Spiders Questionnaire; F-U = Follow Up; GAD = General anxiety disorder; HRV = Heart rate variability; IAPS = International Affective Picture System; iVET = In Vivo Exposure Therapy; IVRE = Immersive Virtual Reality Environments; KIMS-Short = Kentucky Inventory of Mindfulness Skills; LSAS = Liebowitz social anxiety scale; LSAS-SR = Liebowitz social anxiety scale Self-Reported; MAIA = Multidimensional assessment of interoceptive awareness; MBI = Mindfulness Based Intervention; NAS-PI = Novaco Anger Scale and Provocation Inventory; O- AS = Agoraphobic Avoidance Scale; O-BAT = Agoraphobic Behavioral Assessment Task; OCD = Obsessive Compulsive Disorder; OST = One-session treatment; P-ARET = Projection-based augmented reality exposure therapy; PEP - 3 = Psychoeducational Profile, Third Edition; PSA = Public speaking anxiety; PSS = Post-traumatic Stress Symptom Scale; PTSD = Post-traumatic Stress Disorder; RP = Role Plays; RPQ = Reactive-Proactive Questionnaire; SADS = Social avoidance and distress scale; SAS = Spider Anxiety Screening; SBQ = Safety Behaviour Questionnaire-Persecutory Delusions; SDAS = Social Dysfunction and Aggression Scale; STAXI-2 = State-Trait Anger Expression Inventory 2; SUDs = Subject Units of Distress; VAS = Visual Analogue Scale; VAAAT = Virtual Alcohol Approach-Avoidance Training Task; VR = Virtual Reality; VR-CBT = Virtual Reality Cognitive Behavioral Therapy; VRAPT = Virtual Reality Aggression Prevention Therapy; VRE = Virtual Reality Exposure; VRET = Virtual Reality Exposure Therapy; VR= Virtual Reality; VR-CBM-I = virtual reality Cognitive Bias Modification of Interpretations; VRS = avatar that looks like the self; VRN = neutral avatar; WL = Waiting List; YBOCS = Yale-Brown Obsessive Compulsive Scale.

Supplementary Table S2. Quality assessment and risk of bias criteria for observational cohort, case-control, and controlled intervention studies.

CRITERIA	STRONG	MODERATE	WEAK
Research Design	Randomized Control Trial or experimental study	Observational cohort or case-control studies	Uncontrolled studies
Was the research objective clearly stated and directly related to review topic?	Clear description of objective. Outcome measures directly related to topic	Moderately clear, some details missing. Some outcome measures related to topic	Unclear or not stated
Was the study population clearly defined with inclusion/exclusion criteria stated and consistent?	Clear description of population and inclusion/exclusion criteria	Moderately clear, some details missing	Unclear or not stated
Comparator: were the subjects selected from a comparable population in all respects?	Participants were comparable in at least: Diagnosis, age, weight, BMI, severity of disease, timeframe of treatment, similar duration of illness, and number of previous hospitalizations	Participants were comparable in at least three between: Age, sex, schooling, ethnicity, marital status, BMI, diagnosis. Some differences reported in timeframe of treatment, severity of disease, duration of illness, or number of previous hospitalizations.	No comparator group or unclear or not stated
Sample size	>100	50-99	<50
Was the follow-up timeframe sufficient?	1 year or greater follow-up	3 months to 11 months follow-up	< 3 months follow-up
Was treatment thoroughly described?	Clear description of regimen and formula	Moderately clear, some details missing	Unclear or not stated

For outcomes that can vary, did the study clearly define different levels of the outcome?	Clear description, different levels of outcomes reported	Moderately clear description of the different levels of outcomes	No alternate level of outcome reported
Data Collection Method: scale, methods to collect data	Tools are valid and reliable	Tools are valid but reliability not described	No evidence of validity or reliability or not stated
Measurement Bias: Were the outcome measures clearly defined, valid, reliable and implemented consistently?	Valid, reliable, and explained in detail	Measurement valid but reliability not described	Self-reported by participants or not stated or unclear
Selection Bias: Is study sample representative of target population and if <100% eligible cases were selected, were they randomized?	Very likely to be representative of target population, >80% participation rate	Somewhat likely to be representative of target population, 60-79% participation	< 60% participation rate or not stated
Attrition Bias: Was loss to follow-up afterbaseline minimized?	>80% follow-up after baseline	60-79% follow-up after baseline and explanation of those lost	< 60% follow-up after baseline or not reported
Confounders: Were key potential confounding variables measured and adjusted statistically for their impact on the relationship between treatment and outcome?	Confounders identified, discussed, and adjusted for statistically	Confounders identified and discussed	Unclear or not stated

Format modified from National Institutes of Health (2021) [38].

Supplementary Table S3. Assessment of quality and risk of bias of selected experimental studies [38].

**Criteria**	**Anderson et al., 2017 [62]**	**Bosse et al., 2014 [63]**	**Bouchard et al., 2017 [39]**	**Cárdenas-López et al., 2014 [40]**	**Freeman et al., 2022 [43]**	**Gujjar et al., 2018 [44]**	**Gujjar et al., 2019 [45]**	**Hadley et al., 2019 [64]**
Research Design	M	M	S	M	S	M	S	S
Was the research objective clearly stated and directly related to review topic?	S	S	S	M	S	S	S	S
Was the study population clearly defined with inclusion/exclusion criteria stated and consistent?	M	W	S	W	S	S	S	S
Comparator: were the subjects selected from a comparable population in all respects?	W	W	S	W	S	M	M	S
Sample size	W	W	M	W	S	W	W	M

Was the follow-up timeframe sufficient?	W	M	M	N/A	M	M	M	M
Was treatment thoroughly described?	S	S	S	S	S	M	S	M
For outcomes that can vary, did the study clearly define different levels of the outcome?	S	S	S	S	S	M	S	S
Data Collection Method: scale, methods to collect data	M	W	M	S	M	M	M	M
Measurement Bias: Were the outcome measures clearly defined, valid, reliable and implemented consistently?	M	W	M	S	M	M	M	S
Selection Bias: Is study sample representative of target population and if <100% eligible cases were selected, were they randomized?	N/A	S	S	W	S	S	S	S
Attrition Bias: Was loss to follow-up afterbaseline minimized?	N/A	S	M	N/A	S	S	S	S
Confounders: Were key potential confounding variables measured and adjusted statistically for their impact on the relationship between treatment and outcome?	S	W	S	W	S	W	S	S
Total Points	23	25	34	21	36	30	32	33
Overall Score	W	W	S	W	S	M	S	S

**Criteria**	**Jo et al., 2022 [70]**	**Kampman et al., 2016 [46]**	**Kaussner et al., 2020 [47]**	**Klein Tuente et al., 2020 [71]**	**Kim & Lee, 2019 [48]**	**Kim et al., 2020 [49]**	**Malbos, Rapee & Kavakli, 2011 [50]**	**Malbos, Rapee & Kavakli, 2013 [51]**
Research Design	M	S	M	S	M	M	M	M
Was the research objective clearly stated and directly related to review topic?	S	S	S	S	S	S	S	S

Was the study population clearly defined with inclusion/exclusion criteria stated and consistent?	M	S	S	S	M	S	M	S
Comparator: were the subjects selected from a comparable population in all respects?	S	S	M	M	M	S	S	S
Sample size	M	M	W	S	W	M	W	W
Was the follow-up timeframe sufficient?	N/A	M	W	M	N/A	W	N/A	M
Was treatment thoroughly described?	S	S	S	S	S	S	S	S
For outcomes that can vary, did the study clearly define different levels of the outcome?	S	S	M	S	S	S	M	S
Data Collection Method: scale, methods to collect data	M	S	M	S	S	M	S	S
Measurement Bias: Were the outcome measures clearly defined, valid, reliable and implemented consistently?	S	S	M	S	S	M	S	S
Selection Bias: Is study sample representative of target population and if <100% eligible cases were selected, were they randomized?	S	S	M	S	S	M	N/A	S
Attrition Bias: Was loss to follow-up afterbaseline minimized?	N/A	M	S	S	N/A	W	N/A	S
Confounders: Were key potential confounding variables measured and adjusted statistically for their impact on the relationship between treatment and outcome?	S	S	M	S	M	S	W	S
Total Points	29	36	28	37	27	30	21	35
Overall Score	M	S	M	S	M	M	W	S

**Criteria**	**Maples-Keller et al., 2017 [52]**	**Meyerbroeker et al., 2013 [53]**	**Michaliszyn et al., 2010 [54]**	**Miloff et al., 2019 [55]**	**Navarro-Haro et al., 2019 [66]**	**Otkhmezuri et al., 2019 [67]**	**Pot-Kolder et al., 2018 [56]**	**Rus-Calafell et al., 2014 [58]**
Research Design	M	S	S	S	M	M	S	M
Was the research objective clearly stated and directly related to review topic?	S	S	S	S	S	S	S	S
Was the study population clearly defined with inclusion/exclusion criteria stated and consistent?	S	S	S	S	S	M	S	S
Comparator: were the subjects selected from a comparable population in all respects?	S	S	S	S	M	S	S	S
Sample size	S	M	M	S	W	W	S	W
Was the follow-up timeframe sufficient?	N/A	N/A	M	S	N/A	N/A	M	W
Was treatment thoroughly described?	S	S	S	S	S	S	S	S
For outcomes that can vary, did the study clearly define different levels of the outcome?	S	S	S	S	S	S	S	S
Data Collection Method: scale, methods to collect data	M	S	S	S	S	M	M	S
Measurement Bias: Were the outcome measures clearly defined, valid, reliable and implemented consistently?	M	S	S	S	S	M	M	S
Selection Bias: Is study sample representative of target population and if <100% eligible cases were selected, were they randomized?	S	S	S	S	S	N/A	S	N/A

Attrition Bias: Was loss to follow-up afterbaseline minimized?	N/A	W	S	S	N/A	N/A	S	S
Confounders: Were key potential confounding variables measured and adjusted statistically for their impact on the relationship between treatment and outcome?	S	S	S	S	S	S	S	S
Total Points	30	33	37	39	29	24	36	31
Overall Score	M	S	S	S	M	W	S	S

**Criteria**	**Safir et al., 2012 [59]**	**Shiban et al., 2015 [60]**	**Wrzesien et al., 2015 [68]**	**Yuan & Ip, 2018 [69]**	**Zinzow et al., 2018 [72]**
Research Design	M	S	M	M	M
Was the research objective clearly stated and directly related to review topic?	S	S	S	S	S
Was the study population clearly defined with inclusion/exclusion criteria stated and consistent?	S	S	W	S	S
Comparator: were the subjects selected from a comparable population in all respects?	S	M	W	S	S
Sample size	W	W	W	M	W
Was the follow-up timeframe sufficient?	S	M	N/A	N/A	W
Was treatment thoroughly described?	S	S	S	S	S
For outcomes that can vary, did the study clearly define different levels of the outcome?	S	S	S	S	S
Data Collection Method: scale, methods to collect data	S	S	S	N/A	S

Measurement Bias: Were the outcome measures clearly defined, valid, reliable and implemented consistently?	S	S	S	N/A	S
Selection Bias: Is study sample representative of target population and if <100% eligible cases were selected, were they randomized?	S	S	S	N/A	N/A
Attrition Bias: Was loss to follow-up afterbaseline minimized?	S	M	N/A	N/A	S
Confounders: Were key potential confounding variables measured and adjusted statistically for their impact on the relationship between treatment and outcome?	S	S	S	S	S
Total Points	36	34	26	21	31
Overall Score	S	S	M	W	S

Abbreviations. W = weak, M = moderate, S = strong, N/A = not applicable.
